# The immunomodulatory function of adenosine in sepsis

**DOI:** 10.3389/fimmu.2022.936547

**Published:** 2022-07-25

**Authors:** Teng Zhang, Li Yu-Jing, Tao Ma

**Affiliations:** ^1^ Department of General Surgery, Tianjin Medical University General Hospital, Tianjin, China; ^2^ Department of Neurology, Tianjin Medical University General Hospital, Tianjin, China

**Keywords:** adenosine, sepsis, adenosine receptors, immune cells, immunology

## Abstract

Sepsis is an unsolved clinical condition with a substantial mortality rate in the hospital. Despite decades of research, no effective treatments for sepsis exists. The role of adenosine in the pathogenesis of sepsis is discussed in this paper. Adenosine is an essential endogenous molecule that activates the A1, A2a, A2b, and A3 adenosine receptors to regulate tissue function. These receptors are found on a wide range of immune cells and bind adenosine, which helps to control the immune response to inflammation. The adenosine receptors have many regulatory activities that determine the onset and progression of the disease, which have been discovered *via* the use of animal models. A greater understanding of the role of adenosine in modulating the immune system has sparked hope that an adenosine receptor-targeted treatment may be used one day to treat sepsis.

## Introduction

Sepsis is an illness characterized by many potentially fatal organ failures induced by an abnormal host response to infection (1). Early mortality from sepsis has decreased as intensive care management and goal-directed therapies have improved (2). However, the persistent inflammation, immunosuppression, and catabolism syndrome (PICS), which seems to be the leading cause of mortality in sepsis, remains unsolved (3). Increasing evidence currently supports the central involvement of the immune system in sepsis (4). Sepsis occurs when the immune response to invading pathogens fails to restore equilibrium, resulting in a pathological condition marked by prolonged excessive inflammation and immunosuppression (5). As a result, unraveling the complicated process of immune dysregulation in sepsis and developing a tailored immunotherapy has been a focus in the study of sepsis (6). Immune diseases, including immunosuppression in sepsis, are caused by abnormal activation, extensive apoptosis, phenotypic, and functional alterations of immune cells (7). Immunotherapies, such as the administration of IL-7, IL-15, anti-programmed cell death receptor-1 and anti-B and T lymphocyte attenuator, have been demonstrated to reduce mortality due to long-term sepsis (8, 9).

Adenosine is a naturally occurring purine nucleoside that has a relatively short half-life in tissue and important signaling functions (10). A substantial amount of data supports the hypothesis that the capacity of adenosine to modulate the immunological and inflammatory systems by binding to adenosine receptors plays a critical part in health and illness (11). Many novel therapeutic techniques are being developed to regulate adenosine immune functions (12). Adenosine degradation and synthesis inhibitors, and specific agonists or antagonists of diverse adenosine receptor subtypes, are among the pharmacological agents in this class (13–15). We provide a broad overview of adenosine receptors in this review. Separate sections discuss several roles that adenosine plays in determining the function of various cell types thought to be fundamental components of the innate and adaptive immune systems. Lastly, we focus on immunomodulatory role of adenosine in sepsis.

## Immunopathology of sepsis

Sepsis is an abnormal response by the body to an infection (5). Most of the time, the immune system, antibiotics, and control of the source of infection/drainage work together to keep infections under control and eventually cure them, restoring homeostasis (16). However, an infection can turn into sepsis if the body keeps responding in an abnormal fashion (17). Older hypotheses proposed that sepsis progressed from a state of high inflammation to a state of low inflammation to a state of long-lasting and significant immune suppression (1). Systemic inflammatory response syndrome (SIRS) and compensatory anti-inflammatory response syndrome (CARS) are two terms that jointly describe this paradigm (18). Recent studies have shown that inflammation and reduced immunity happen simultaneously, not sequentially (5, 18, 19). During the early stages of an infection, both pro-inflammatory and anti-inflammatory cytokine storms occur (5, 20). Analysis of the expression of leukocyte genes in patients with severe sepsis has shown that the inflammatory response and the expression of genes related to immunosuppression occur, immediately after the onset of sepsis (19). It is also thought that sepsis is synonymous with PICS (21). However, an integrated viewpoint holds that SIRS and its opposite, CARS, do not occur separately but simultaneously, which is an interesting idea (5).

Infections trigger excessive inflammatory responses, which may result in abnormal complement and coagulation system activation, as well as vascular endothelial dysfunction. Complements C3a and C5a have significant pro-inflammatory features, including the ability to recruit and activate leukocytes and platelets (22). Prolonged coagulation system activation may result in diffuse intravascular coagulation (23). Additionally, prolonged inflammation causes tissue damage and the production of damage-associated molecular patterns, which results in greater activation of the immune system, ultimately leading to organ damage and malfunction. Persistent, severe inflammation induces widespread death of immune cells (particularly lymphocytes and dendritic cells (DCs)), while retarding neutrophil apoptosis (9). However, these neutrophils have diminished bactericidal activity and produce fewer cytokines. CD4+ T cells undergo programmed cell death 1, and the fraction of regulatory T (Treg) cells increases, impairing effector T cell activity (9). In addition, monocytes and macrophages show a decreased capacity to generate several pro-inflammatory cytokines in response to lipopolysaccharide (LPS) activation, a phenomenon known as “immuno-paralysis” (9). These factors all contribute to significant immunosuppression in sepsis, particularly during the latter stages, increasing the risk of secondary infection (16). In summary, immune dysfunction is important for the development and progression of sepsis. Thus, immunotherapy will become a critical component of sepsis treatment.

## Adenosine and adenosine receptors

Adenosine is released constitutively from a variety of cell types under normal circumstances, and extracellular quantities are in the nanomolar range (24). Inflammation and tissue damage significantly increase adenosine release and synthesis, and tissue levels may increase a hundred-fold (13). Adenosine is not only a metabolic product of ATP but also a substrate for its production. Intracellular adenosine is regulated *via* the soluble intracellular 5’-nucleotidase CD73. CD73 also acts as an ectoenzyme that works in conjunction with CD39 to degrade ATP to adenosine in the extracellular environment. After it is synthesized, adenosine would be swiftly decomposed into inosine by adenosine deaminase or phosphorylated to AMP by adenosine kinase, giving it a biological half-life of less than ten seconds (25).

**Figure 1:**
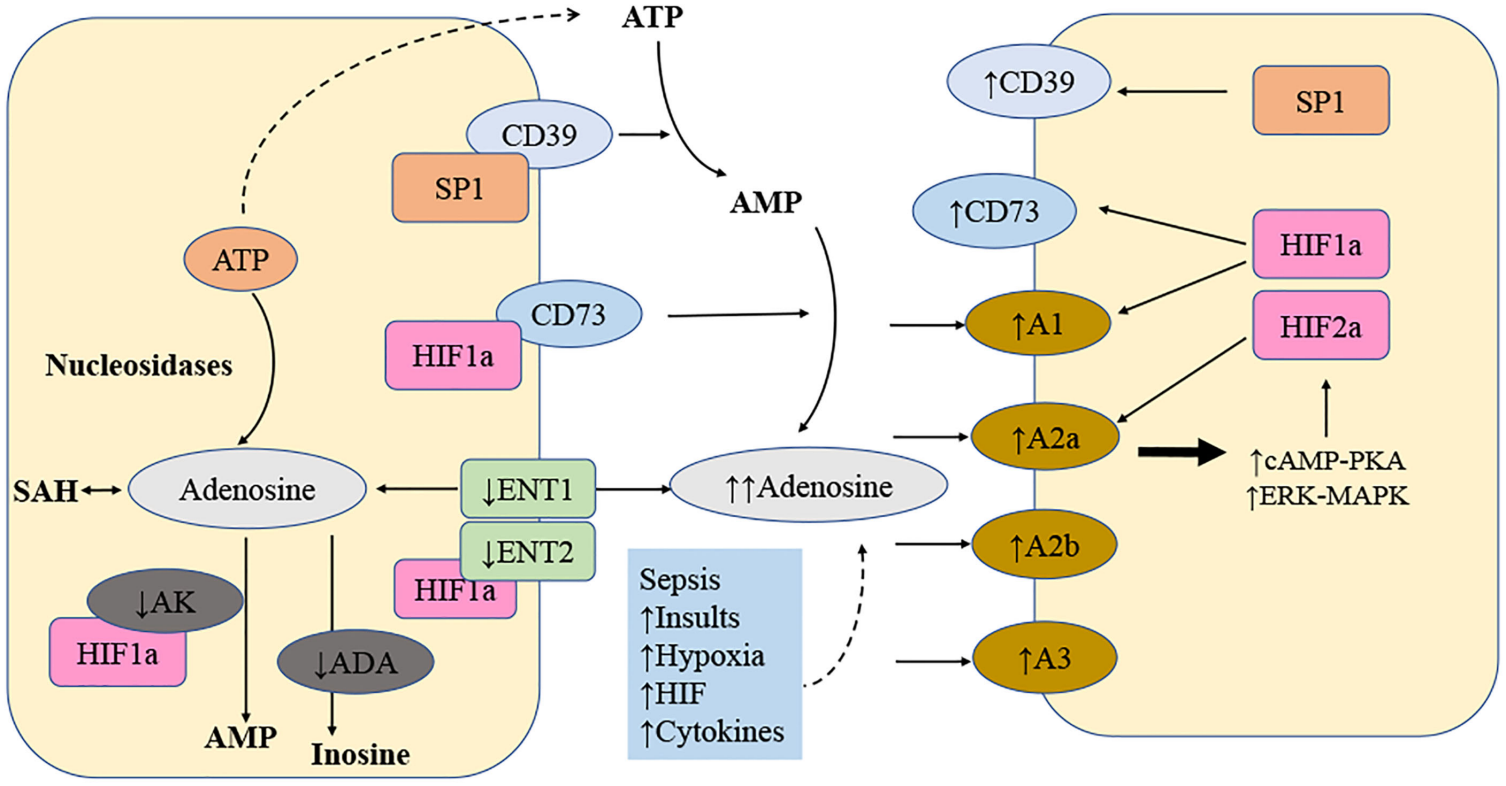
Local amplification of adenosine signaling in sepsis. The levels of extracellular adenosine is influenced by several factors. Adenosine is transferred by a transporter called equilibrate nucleoside transporter 1 (ENT1) and many additional transporters. Numerous mechanisms result in the release of ATP. ATP is converted to adenosine by the combination of ectonucleoside triphosphate diphosphohydrolase 1 (ENTPD1; also known as CD39) and ecto5′-nucleotidase (NT5E; also known as CD73). After that, adenosine could be converted to inosine, adenosine monophosphate (AMP), or S-adenosylhomocysteine (SAH). An increase in extracellular adenosine concentration from the baseline (20-300 nM) to up to 30 mM dramatically enhances sepsis-related adenosine signaling. Additionally, there is a strong simultaneous increase of enzymes involved in the metabolic process that degrade ATP into adenosine and inhibition of enzymes to degrade adenosine, for example, adenosine kinase (AK). The following variables regulate adenosine signaling in diseased conditions: more ATP released into extracellular space as precursor of adenosine; more ENTPD1 and NT5E expression; HIF2 induce more A2a receptor expression; HIF1 induce more A2b receptor expression; HIF1 inhibit AK, ENT1 and ENT2. ADA stands for adenosine deaminase; cAMP stands for cyclic AMP; ERK stands for extracellular signal-regulated kinase; MAPK stands for mitogen-activated protein kinase; and PKA is for protein kinase A.

Adenosine elicits physiological responses by binding to one or more of the four transmembrane adenosine receptors (A1, A2a, A2b, and A3) (**Table 1**). A substantial amount of data indicates that the adenosine receptors regulate cellular activity *via* their association with the G proteins, although some effects have been described in the absence of the G proteins (27, 40, 41). Historically, it was believed that adenosine receptor signaling occurred *via* the activation or inhibition of adenylyl cyclase, accompanied by a change in the intracellular concentration of cyclic AMP (cAMP). Adenosine receptors were first classed as A1 or A2 based on their capacity to inhibit or stimulate cAMP buildup (26). Subsequent investigations refined the categorization of the adenosine receptors, and the cAMP-increasing A2 receptors were classified as high-affinity (A2a) or low-affinity receptors (A2b) (33). Similar to the A1 receptors, the A3 receptors have the ability to regulate mast cell degranulation *via* reducing the intracellular cAMP concentration (34). In addition to the adenylyl cyclase–cAMP system, adenosine receptors may be associated with a variety of different pathways (**Figure 1**).

**Table 1:** Characteristics of adenosine receptors.

Name	A1	A2a	A2b	A3
**Human chromosome gene location**	1q32.1	22g11.2	17p11.2–12	1p21-p13
**G protein coupling**	Gi (26)	Gs (27)	Gs, Gq (28)	Gi, Gq (29)
**Adenosine affinity**	1–10nM	30nM	1,000nM	100nM
**Effector system**	Inhibit: Adenylyl cyclase cAMP system (26); Ca2+ channels (30) Enhance: Phospholipase C (26); K+ channels (31); PI3 kinase; MAP kinase (32)	Enhance: Adenylyl cyclase cAMP system (26); MAP kinase (27)	Enhance: Adenylyl cyclase cAMP system (33); Phospholipase C; MAP kinase (28)	Inhibit: Adenylyl cyclase cAMP system (34) Enhance: Phospholipase C (29); PI3 kinase; MAP kinase (35)
**Clinically approved therapy**	Agonist: Paroxysmal supraventricular tachycardia (Adenosine) (36) Antagonist: Asthma (Theophylline) (37)	Agonist: Myocardial perfusion imaging (Adenosine) (38) Antagonist: Parkinson’s disease (Istradefylline) (39)		

Traditionally, activation of the A1 receptor was associated with Gi-mediated suppression of adenylyl cyclase (26). It is currently recognized, however, that it is also associated with a variety of kinase pathways, such as mitogen-activated protein (MAP) kinase, protein kinase C and phosphoinositide 3 (PI3) kinase (32). Furthermore, ion channels can be influenced by the A1 receptor. For example, the A1 receptor activates K+ channels (31) and inhibits Ca^2+^ channels (30).

While the Gs-protein-coupled receptor A2a mainly signals through the normal adenylate cyclase–cAMP/protein kinase A (PKA) pathway, it may also signal directly *via* cAMP (27). The signaling pathway downstream of PKA is activated when the cAMP responsive element binding protein (CREB) is phosphorylated (42). There are two ways in which activated CREB might affect gene expression: directly by binding to a promotor or indirectly by competing for a necessary cofactor, such as with nuclear factor-κB (27). The A2b receptor may activate adenylyl cyclase *via* Gs, simultaneously activating phospholipase C through Gq (28). Some interaction between these two systems seems to occur. Both are required for the human mast cell activation-induced increase of IL-4 production (43).

Adenylyl cyclase inhibition through Gi and PLC activation *via* Gq are the most common signaling pathways linked with A3 receptor activation (29). Additionally, the A3 receptors may affect cellular activity through the MAP kinase, and PI3 kinase pathways. Indeed, the disruption of the Gi proteins or the inhibition of PI3 kinase inhibits the A3 receptor-dependent increase of histamine release in mast cells (35).

## Adenosine in sepsis

Adenosine is required for the suppression of an immunological response. Adenosine levels in the extracellular space quickly increase in response to systemic inflammation or tissue damage (44). Plasma adenosine concentrations were observed to rise tenfold in septic shock patients (45, 46). This increase was explained by reduced adenosine deaminase and adenosine kinase activity and an increase in the activity of CD73 (47). The immunosuppressive properties of adenosine are mostly regulated by the A2a receptors. Endotoxin or inflammatory mediators rapidly increase A2a and A2b receptor expression (48). It has been shown that activating the A2a receptor dramatically reduces tissue injury in systemic inflammation (49). Although inhibiting extreme immune cell activation through the A2a or A2b receptors would be advantageous during the early stage of sepsis or endotoxemia, the same receptors may also cause immunosuppression (50). In a chronic model of polymicrobial sepsis, inhibiting A2a receptor signaling boosted survival by enhancing bacterial clearance, lowering IL-10 release, and maintaining lymphocyte function (51). Conversely, activating the A1 or A3 receptor would be advantageous in sepsis by decreasing mortality and renal and hepatic injury (52, 53).

## Effects of adenosine on immune cells

Adenosine, which is produced by both immune and non-immune cells, is involved in the immune modulation of several immune cells and may contribute to the pathophysiology of sepsis by mediating immune suppression.

### Adenosine and macrophages

The effects of adenosine on macrophage-derived cytokines has garnered substantial interest, because macrophage-generated cytokines are critical initiators of immunological responses (54). An impressive amount of information has been amassed about the decrease of tumor necrosis factor (TNF) production by adenosine receptor activation subsequent to macrophage activation because TNF was one of the first cytokines discovered (55). A2a receptors are present on macrophages, and their activation may inhibit the production of pro-inflammatory cytokines (56). Activation of the A2a receptor on macrophages inhibits the production of TNF, as shown in studies using knock-out mice (57). Additionally, the use of specific A2b receptor antagonists in Adora2a-/- mice revealed that the A2b receptor plays a role in suppressing TNF release (57). Because the deletion of Adora2b has no impact on TNF production in the presence of intact A2a receptors, A2b receptors are only effective when their activity is not masked by A2a receptors (58).

A similar picture is developing about the way stimulation of the adenosine receptor augments IL-10 production. As established in research using the RAW264.7 macrophage cell line, A2a receptors play a crucial role in increasing the production of IL-10 from macrophages (59). Similarly, investigations in knockout mice indicated that A2a receptors are necessary for the stimulation of IL-10 secretion in *Escherichia coli* exposed macrophages (60). Additionally, A2b receptors have been involved in post-transcriptional pathways that enhance the release of IL-10 from LPS-stimulated macrophages (58). While A2a receptors also play a role in this process, the activation of macrophage A2b receptors results in the anti-inflammatory M2 phenotype (61).

### Adenosine and DCs

Adenosine receptors are expressed on the surface of DCs, and their activation may modulate the immunological response during sepsis. A2a receptors are found on myeloid and plasmacytoid DCs and their activation may result in a change in the cytokine profile from pro- to anti-inflammatory, possibly characterized by increased IL-10 secretion and decreased IL-12, IL-6, and IFN-γ secretion (62). This alteration in the cytokine profile results in the preferential development of naive CD4+ T cells to T-helper 2 (Th2) cells rather than Th1 cells (63). In animal tests, giving DCs that had been treated with an A2a receptor agonist helped protect mice from damage caused by ischemia and reperfusion by stopping IFN-γ production (64). When A2b receptors are activated on hematopoietic progenitor cells in mice, DCs differentiate into a distinct phenotype characterized by the concurrent expression of monocyte and DC surface markers (65). These cells exhibit the DC-specific marker CD209, have little or no expression of the DC marker CD1a, and fail to shed the monocytic marker CD14 (66). Unlike normal myeloid DCs, adenosine-differentiated DCs have impaired activity (66). Additionally, activation of the A2b receptor on DCs boosted IL-6 production, resulting in greater Th17 polarization of naive T cells (67). A1 receptor activation reduces resting DCs vesicular MHC class I cross presentation, suggesting that A1 receptors may be involved in DC maturation (68). Similarly, A3 receptor activation was demonstrated to decrease the production of IL-6 and TNF to exert anti-inflammatory actions (62). Another study discovered that A3 receptors agonists protect endo-toxemic mice by reducing IL-12 and IFN-γ levels (47). In summary, the existing evidence suggests that adenosine has a dual role in regulating DCs activity and, through the A1 or A3 receptors, increases the migration of immature DCs to sites of inflammation. Adenosine induces an anti-inflammatory DCs phenotype through A2a receptors, hence directing T-cell responses toward a Th2 profile.

### Adenosine and neutrophils

**Figure 2:**
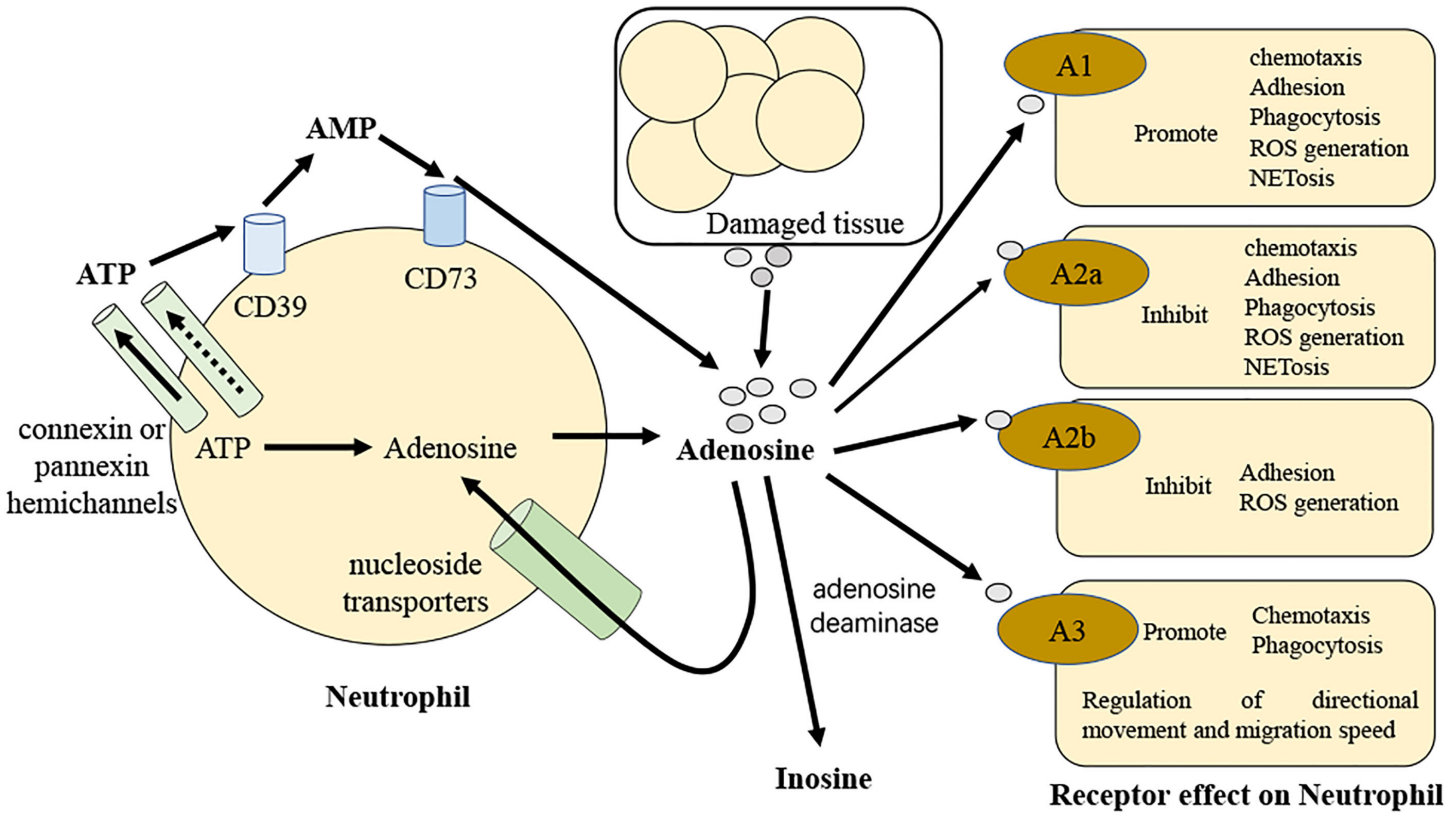
Diagrammatic over view of adenosine regulation of neutrophils in sepsis. Adenosine and its precursors are released by activated neutrophils. These precursors can be degraded to adenosine so that they may then operate autocrine to control neutrophil activity. Along with adenosine, neutrophils release ATP in response to stimulation *via* connexin or pannexin hemichannels and this is promptly degraded to adenosine by the neutrophil surface-expressed ectoenzymes CD39 and CD73. In sepsis, neutrophils and adenosine talk to each other by binding to G protein-coupled receptors A1, A2a, A2b, and A3.

As a major modulator of neutrophil activity, adenosine controls the production of reactive oxygen species by neutrophils as well as their capacity for phagocytosis (69). Adenosine has a concentration- and receptor-dependent effect on neutrophil phagocytosis. At pico- to nanomolar doses of adenosine, activation of the A1 receptor promotes FcR-mediated phagocytosis in human neutrophils (70, 71). On the other hand, micromolar dosages of adenosine or an A2aR agonist impair phagocytosis (72, 73). Adenosine receptors have not been widely studied in subsequent research on neutrophil phagocytosis. It is likely that low amounts of adenosine stimulate phagocytosis by binding to A1 receptors, but high quantities of adenosine restrict phagocytosis by activating the A2a receptors (69) (**Figure 2**).

Several chemokines, cytokines, and lipid mediators generated from arachidonic acid are released by active neutrophils, each having a unique effect on the ongoing inflammatory response. Activation of the adenosine receptor inhibits pro-inflammatory mediator release from active neutrophils while boosting anti-inflammatory mediator release. Through the A2a receptors, adenosine suppresses neutrophils production of TNF and several macrophage inflammatory proteins (74). The A1 agonist 2-chloro-N6-cyclopentyladenosine and the dual A2a/A2b agonist 5’-(N-cyclopropyl)-carboxamido-adenosine both inhibit TNF production from LPS activated neutrophils, with the A2 agonist being 1000-fold more potent than the A1 agonist (75). Similarly, CGS-21680 reduced the production of TNF by neutrophils activated with LPS by activating the A2a receptor (76). Arachidonic acid is converted to prostaglandin E2 (PGE2) or thromboxane A2 (TXA2) by the induction of COX-2 activity (77). PGE2 is an anti-inflammatory molecule that suppresses neutrophil aggregation, chemotaxis, and the production of superoxide (78). Differently, the pro-inflammatory mediator TXA2 promotes platelet aggregation and coagulation (79). The capacity of mice lacking the A2a receptor (Adora2a-/-) to activate COX-2 in leukocytes is reduced (80). Additionally, the activation of the A2a receptors on human neutrophils activated with fMLP enhances COX-2 induction and boosts PGE2 synthesis without decreasing TXA2 synthesis (81). Through the 5-lipoxygenase (5-LO) route, arachidonic acid can also be transformed into leukotriene B4 (LTB4), which is a powerful chemoattractant for neutrophils, with the potential to trigger an oxidative burst and degranulation. Previous investigation has shown that the production of LTB4 in whole blood is inhibited by adenosine analogs when fMLP is used (75). Further work separated active neutrophils and revealed that eliminating endogenous adenosine or inhibiting the A2a receptors increased LTB4 synthesis (82). Similarly, until adenosine is withdrawn or an A2a antagonist is administered, active neutrophils cannot convert arachidonic acid to 5-LO products (83). Taken together, these findings indicate that activating the A2a receptors on neutrophils has the ability to affect the release of inflammatory mediators.

The post-capillary venular endothelium attracts neutrophils to inflammatory sites from the circulation by modifying the expression of sticky molecules on its surface. Adenosine inhibits neutrophil attachment to the endothelium through the A2a receptors by reducing the quantity and function of the adhesion molecules (84). By contrast, the A1 receptors promote neutrophil adhesion by enhancing a range of sticky molecules present on the endothelium (85). Neutrophils migrate along chemoattractant gradients within the tissue. Chemoattractants include activated complement components (C5a), bacterial products, and chemokines. Through the A1 and A3 receptors, adenosine has been demonstrated to stimulate neutrophil migration (86). Neutrophils may gather A3 receptors locally on the cell membrane in the forward direction, enabling them to migrate in a particular direction (87).

The ability of adenosine to control a neutrophil oxidative burst is one of the most well-known effects of adenosine on cells. Micromolar concentrations of adenosine inhibit nearly 50% of oxidative burst activity in fMLP-stimulated neutrophils (88). The neutrophil oxidative burst has been connected to impact of adenosine on neutrophils *via* the A2a receptor, which has been shown to be inhibited by A2a agonists in response to stimuli such as fMLP and TNF (69, 89). The A2b receptor agonist reduced superoxide production by around 50% in fMLP-stimulated neutrophils, a result not seen in A2b-deficient mice (90). On the other hand, the A1 receptor agonist CPA caused more superoxide to be made when human neutrophils were activated by FcR. The adenosine antagonist 8-(p-Sulophenyl) theophylline stopped this from happening (70, 91). A detailed examination of A3 agonists and antagonists revealed that, although A3 receptors may contribute to the suppression of the oxidative burst, the inhibition is primarily mediated by A2a activation (92, 93).

Cell death is necessary for neutrophil balance in the resting state and infection. Neutrophils die through a variety of different apoptosis subprocesses, necrosis and as a consequence of NET formation (‘NETosis’) (94, 95). Numerous investigations have indicated that adenosine prevents resting human neutrophils from undergoing apoptosis *via* the adenosine 2a receptor (96). In mice with systemic inflammatory response syndrome, activating the adenosine 2a receptor stops neutrophils from dying in a manner that depends on autophagy (97). Neutrophil extracellular traps (NET) were found to be extracellular strands of decondensed DNA bound to histones and granule proteins, which were released from dying neutrophils to catch and kill microorganisms. Signaling through the A2a receptor subtype, adenosine has a strong effect on neutrophils from a large number of healthy human donors to strongly stop NET. The adenosine 2a receptor agonist CSG21680 had the same effect on NETs as adenosine, while the adenosine 2a receptor antagonist ZM241385 blocked the effects of adenosine on NETs (98).

### Adenosine and lymphocyte

**Figure 3:**
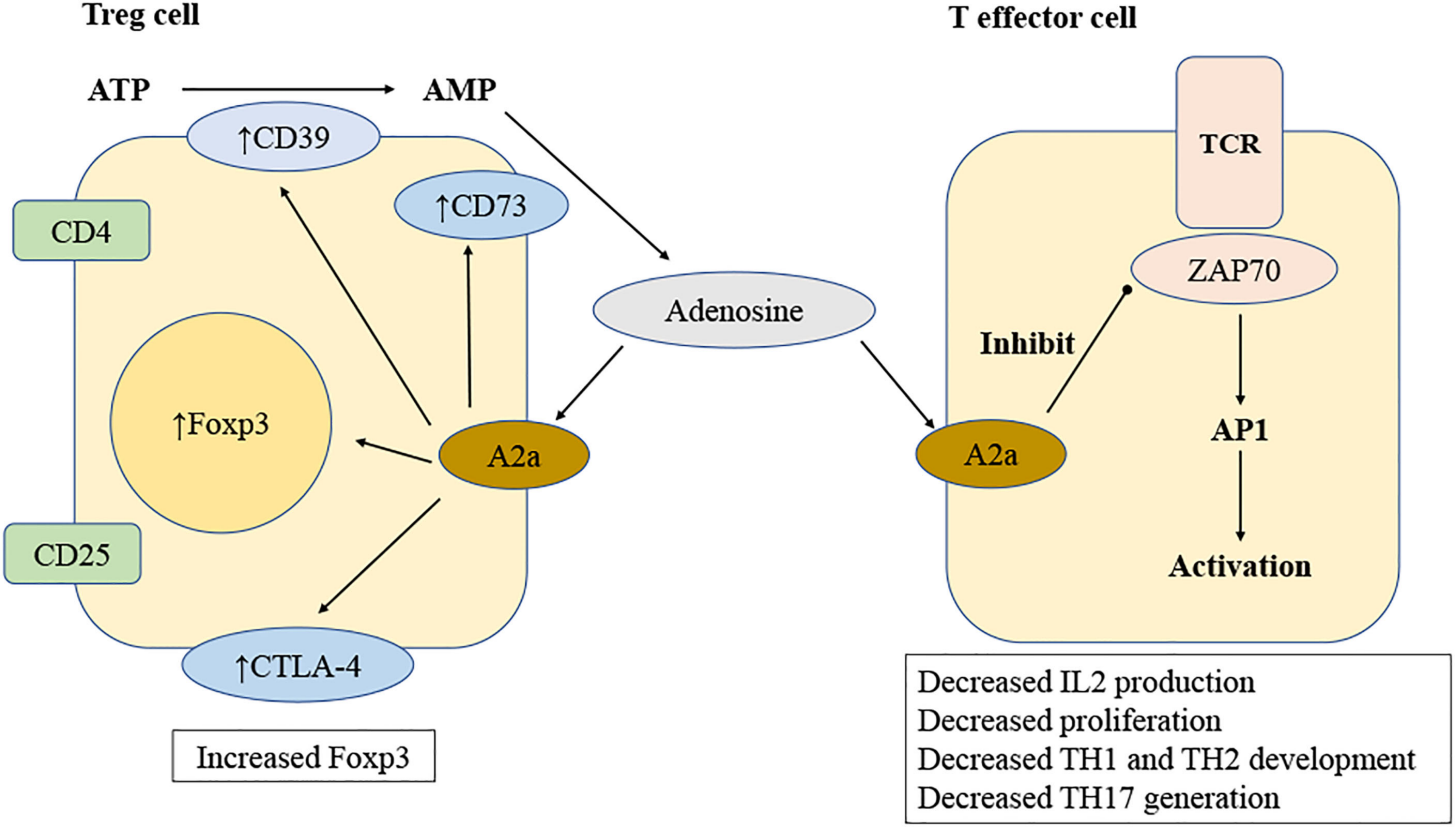
Mechanisms of adenosine regulation of T lymphocyte. Adenosine is produced by regulatory T (Treg) cells when ATP is degraded sequentially by CD39 and CD73. Adenosine stimulates T-effector cells *via* A2a receptors, therefore inhibiting T cell receptor (TCR) signal by inhibiting ZAP70 phosphorylation and activating the transcription factor activator protein 1(AP-1). Reduced TCR signal results in lower synthesis of IL-2 and expression of CD25, leading to inhibited T cell proliferation. Activating A2a receptors changes the development of T helper 1 (TH1), TH2 and TH17 lymphocytes. The A2a receptor activation of T cells results in an increase CTLA-4, PD-1 and Foxp3 expression.

Adenosine may directly change lymphocyte responses by binding to and activating adenosine receptors on lymphocytes. This is different from the manner in which it affects lymphocyte function indirectly by activating adenosine receptors on innate immune cells such as DCs (99). Numerous studies have examined the influence of adenosine receptor activation on many lymphocyte activities utilizing adenosine receptor knockout mice (100–103). Several studies, including those mentioned above, have shown that A2a receptors are crucial in modulating lymphocyte responses (104–106) (**Figure 3**).

Through the A2a receptors, adenosine inhibits IL-2 production of naive CD4+ T cells, hence inhibiting T lymphocyte proliferation in response to T-cell receptor activation (105). A2a receptor activation can also cause an increased expression of negative co-stimulatory molecules (107) such as PD-1 and cytotoxic T lymphocyte antigen 4 (CTLA-4), decrease the expression of CD40L, and stop the release of IFN-γ and IL-4 (108). When the A2a receptor on T cells is turned on, both differentiation into Th1 and Th2 cells and cell death caused by activation simultaneously cease (106).

As with CD4+ cells, adenosine suppresses the generation of IL-2 by both type 1 cytotoxic T (TC1) and type 2 cytotoxic T (TC2) CD8+ cells with the action being believed to be mediated *via* the A2a receptor (109). However, activation of the A2a receptor did not affect the generation of TC1 (IFN-γ) or TC2 (IL-4 and IL-5) cytokines in one study; additionally, the pharmacological stimulation of the A2a receptor had no impact on the cytolysis activity of TC1 or TC2 cells (110).

Recent research has demonstrated that adenosine plays a critical role in modulating the immunological suppressive features of Treg cells, which may play a crucial role in maintaining immune balance and avoiding excessive tissue damage. Treg cells, characterized as CD4+/CD25+/Foxp3+ T lymphocyte and their capacity to inhibit CD4+/CD25- lymphocyte proliferation, were discovered to display high amounts of CD39 and CD73 (111). Recent findings demonstrating that Foxp3+ really induces CD39 expression revealed a molecular relationship between the transcription factor Foxp3+ and CD39 (112). It was shown that Treg cells may hydrolyze exogenous ATP effectively to create adenosine through CD39 and CD73. In another study, the ability of Treg cells to synthesize adenosine was important for their regulatory function. Treg cells from CD39-deficient mice lost their ability to inhibit the growth of CD4+/CD25- lymphocyte, but this could be mitigated by adding soluble exogenous CD39 (112). When A2a-receptor-deficient target cells (CD4+/CD25-) were mixed with wild-type Treg cells, they grew faster than wild-type cells. This showed that the A2a receptor has a key role. Additionally, CD4+/CD25- lymphocyte showed an increase in A2a receptor expression by day 4, which is the peak time for Treg-cell-mediated target cell proliferation inhibition. This shows once again that adenosine production is a critical component of the Treg-cell armory (113). Similar to target cells Treg cells contain A2a receptors, and stimulation of these receptors induces the production of Foxp3+ in the Treg cells (114). The A2a receptors on Treg cells were proven to have functional importance in a mouse model of colitis and allergy (115). In this setting, it was discovered that adoptively transplanted Treg cells deficient in A2a receptors were unable to suppress colitis (116). Thus, ectoenzymes in Treg cells create adenosine, and A2a receptors in Treg cells are essential for their immunosuppressive function.

## Overall effect of adenosine receptors in sepsis

Numerous investigations have shown the relevance of multiple adenosine receptors in sepsis utilizing both genetic and pharmacological approaches (51, 117, 118). Both A2a receptor deletion mice and ZM241385 pharmacological antagonists reduced cecal ligation and puncture (CLP) induced mortality through a mechanism related to a reduced bacterial burden (51, 118). Intriguingly, when paired with antibiotics, activation of the A2a receptor protected against sepsis created by *E. coli* injection, probably by inhibiting an excessive inflammatory reaction caused by the rapid drug-mediated death of vast numbers of bacteria (60). Additionally, it was shown that antagonistic A2a receptors provided protection against sepsis-induced lymphopenia (119). In polymicrobial sepsis produced by CLP, blockade of the A2b receptor has been found to increase survival by enhancing bacterial phagocytosis by macrophage (120). An increase in mortality in the CLP model was related to an increase in hepatic and renal damage generated by inflammation when the A1 receptors were blocked in a separate study (52). Additionally, it was also shown that the A1 receptor antagonist L-97-1 protects against renal impairment and improves survival after sepsis (121). Experimental investigations have indicated that A3 receptor activation may reduce renal and hepatic damage in mice caused by CLP sepsis, resulting in a decrease in mortality (53). Adenosine receptors are found on a wide range of cells and exert several physiological functions in the human body. While activation of the A1 receptor may have deleterious cardiovascular and pulmonary consequences, stimulation of the A3 receptor seems to be harmless (122). In summary, A2a receptor blockage and A3 receptor stimulation in animal models of sepsis show that selective A2a receptor antagonists and selective A3 receptor agonists have great potential for application in sepsis treatment.

## Conclusions and perspectives

The adenosine receptor system developed as a rapid sensor of tissue damage as well as the primary ‘first-aid’ mechanism for tissues and organs. Thus, activation of adenosine receptors retain tissue function and protects it from additional harm after an acute injurious shock. The ability of the adenosine receptor system to guard against acute shocks may be eclipsed by its impaired ability to protect against chronic assaults. Additionally, some chronic disease conditions such as asthma may worsen tissue dysfunction through the adenosine receptor system.

Recent advances in our understanding of the different adenosine receptors and the complex manner in which cells respond to adenosine have helped us find novel pharmacological targets for reestablishing tissue function in a variety of diseases. Preclinical research using both deletion and pharmacological techniques has shown that the many adenosine receptors are important in how the body reacts to sepsis.

While adenosine receptor agonists have potent immunomodulatory properties, their widespread tissue distribution may restrict their use when treating inflammatory disorders. Adenosine receptor antagonists are more selective. Adenosine tends to accumulate at the location of the damage. As a result, adenosine antagonists have a better chance for clinical application. In a similar fashion, inhibiting the enzymes and transporters involved in the buildup of extracellular adenosine enables local targeting of adenosine receptors. It is obvious that a greater knowledge of adenosine receptor activity is necessary before the immense promise of adenosine-based therapeutics to alleviate human suffering can be fulfilled.

## Author contributions

TZ contributed to collection of references and manuscript preparation. TM and LY-J contributed to manuscript modifications. All authors contributed to the article and approved the submitted version.

## Funding

This work was supported by grants from National Natural Science Foundation of China (82172122 to MT), Tianjin Municipal Health Commission (RC20145 to ZT). This work was also funded by Tianjin Key Medical Discipline (Specialty) Construction Project.

## Conflict of interest

The authors declare that the research was conducted in the absence of any commercial or financial relationships that could be construed as a potential conflict of interest.

## Publisher’s note

All claims expressed in this article are solely those of the authors and do not necessarily represent those of their affiliated organizations, or those of the publisher, the editors and the reviewers. Any product that may be evaluated in this article, or claim that may be made by its manufacturer, is not guaranteed or endorsed by the publisher.
